# Fungating scrotal mass: A rare clinical presentation of testicular tumor

**DOI:** 10.4103/0970-1591.42627

**Published:** 2008

**Authors:** B. S. Yadav, S. Ghoshal

**Affiliations:** Department of Radiotherapy, Post Graduate Institute of Medical Education and Research, Chandigarh, India

**Keywords:** Fungating mass, scrotum, testicular tumor

## Abstract

A 20-year-old male patient presented with fungating scrotal mass. Investigations revealed yolk sac tumor with lung metastasis. The patient was treated with systemic chemotherapy. There was complete disappearance of the scrotal mass as well as metastatic disease from the lung. Fungating scrotal mass is a rare presentation of testicular tumor. This rare presentation is reported here. This is second such case in the English literature.

## INTRODUCTION

The classic presentation for testicular tumors is that of a healthy male in the third or fourth decade of life with a painless, swollen, and hard testis. The presentation can vary with the amount of disease, clinical stage, and the presence of metastasis at the time of referral. Fungating scrotal mass due to testicular tumor is a very rare presentation. Management of such a presentation is discussed here.

## CASE REPORT

A 20-year-old male presented in radiotherapy out patient department, with complaints of swelling left testis since 6 months. He was operated 6 months back in a local hospital, where left scrotal orchiectomy was done. The patient did not receive any radiation or chemotherapy after orchiectomy. The swelling recurred with in 2 months and developed in to a fungating mass with foul-smelling discharge.

**Figure 1:**
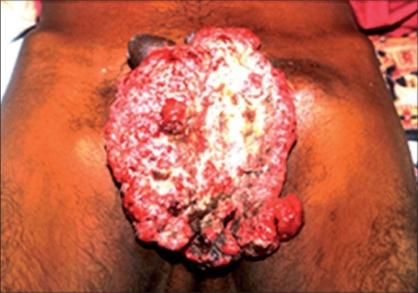
A large 15×10×5 cm cauliflower like fungating growth arising from the scrotal region covering penis and pushing it to the right side. The scrotal skin is covered by the mass. The mass is reaching up to anterior abdominal wall in its lower part. There are areas of slough and bleeding from the mass

On local examination, there was a 15 × 12 × 10 cm^3^ cauliflower-like fungating growth arising from the scrotal region covering penis and going up to anterior abdominal wall in its lower part [Figure 1]. It was pushing penis superiorly and to the right side. Scrotum was totally covered by this mass. Bilateral inguinal lymph nodes were enlarged, the largest one measuring 5 × 5 cm on the left side and 3 × 3 cm on the right side.

**Figure 2:**
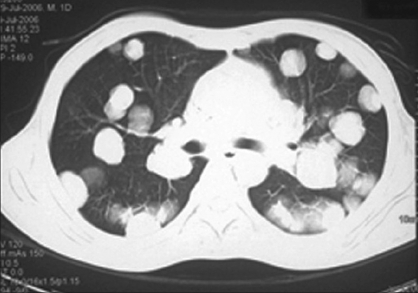
Contrast-enhanced computerized tomography of the chest showing multiple ill-defined nodules (1-3.5 cm) in both the lung fields. Some of them are pleural based and peripherally located. Many of the nodules are showing feeding vessel sign suggestive of metastases

**Figure 3:**
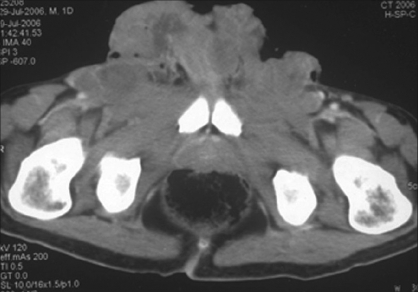
A large 16×14×5 cm mass lesion arising from the scrotum it is irregular in out line and heterogeneous in enhancement. It is reaching up to the level of lower anterior abdominal wall with parietal wall infiltration. The penis is deviated to the right side and is encased by the mass. There are bilateral conglomerate inguinal lymph nodes (3-4 cm)

α-Fetoprotien was 4.4 ng/mL and β-HCG was 6.8 miU/mL. However, LDH was 5380 U/L. Chest X-ray showed multiple canon ball shadows in bilateral lungs suggestive of metastases. Contrast-enhanced computerized tomography (CECT) of the chest showed multiple ill-defined nodules (1-3.5 cm) in both the lung fields. Some of them were pleural based and peripherally located. Many of the nodules were showing feeding vessel sign suggestive of metastases [Figure 2]. The CECT of abdomen did not reveal any significant retroperitoneal lymphadenopathy. There was a large 16 × 14 × 5 cm^3^ mass lesion arising from the scrotum. It was irregular in outline and heterogeneous in enhancement. It was reaching up to the level of lower anterior abdominal wall with parietal wall infiltration. The penis was deviated to the right side and was encased by the mass. There were bilateral conglomerate inguinal lymph nodes (3-4 cm [Figure 3]). Biopsy from the scrotal mass revealed yolk sac tumor.

The patient was started on combination chemotherapy with Bleomycin, Etoposite, Cisplatin (BEP) regimen. The mass regressed rapidly due to chemotherapy, locally as well as in the chest leading to complete response. The inguinal nodes also regressed completely. Postchemotherapy tumor markers were with in normal limits. After completion of chemotherapy scrotum was reconstructed by plastic surgeon. The patient is on regular follow-up with no evidence of disease locally as well as in the lungs. On every follow-up, patient was examined clinically, and chest X-ray and USG abdomen were done at completion of treatment and at 4 months. All the tumor markers were normal on follow-up.

## Discussion

The incidence of testicular tumors is 2-3/100,000. Scrotal mass is the commonest presentation of testicular tumors. The involvement of the skin is rare, possibly due to tough investing tunica albuginea.[1] Hyouchi et al.[2] have reported a giant testicular mass causing scrotal gangrene. This was possibly due to direct infiltration by the tumor. The patient had recurrence in the left scrotal sac with direct extension through the orchiectomy scar. Our patient also underwent scrotal orchiectomy in a private hospital where possibly the seedling occurred during surgery leading to such a huge fungating growth with in 2 months through the orchiectomy scar.

On literature review, it was found out that this is the second case of fungating scrotal mass due to a testicular tumor being reported in the English literature. First case was also reported from India by Nabi *et al.*[3] in the year 2002. The cause for such advanced presentation is due to ignorance on the part of the patient to report late to the tertiary hospital. Some times the patient have financial or family problem leading to such delay. This is an unusual presentation due to testicular tumor, with a fungating mass and diffuse involvement of scrotum and bilateral inguinal lymph nodes. The surgeons should proceed with high-inguinal orchiectomy rather than going for scrotal approach to avoid such complication. Although scrotal involvement due to testicular tumor with inguinal nodes is an ominous sign, this was not true in the present case. There was complete response to chemotherapy.

Testicular tumor should be always be kept in the differential diagnosis of fungating scrotal masses, as availability of an effective chemotherapy has improves prognosis of disease even in advanced stages. These tumors respond dramatically to combination chemotherapy with BEP regimen as also seen in our case. These tumors are very sensitive to chemotherapy as well as radiation therapy. Although rare, this case highlights the importance of testicular self-examination and a supportive patient-provider relationship in early diagnosis and improved outcome. All attempts should be made for early diagnosis and treatment of this condition to avoid mutilating surgery. All these tumors respond dramatically to systemic combination chemotherapy. This facilitates less mutilating surgery with no requirement of flap reconstruction and preservation of urethra.
